# Seroprevalence and factors associated with SARS-CoV-2 infection among education workers after the first wave: the first cross-sectional study in Brazil

**DOI:** 10.1590/0037-8682-0606-2021

**Published:** 2022-04-29

**Authors:** Pâmela de Castro França, Paulo Goberlânio Barros Silva, Jose Lima de Carvalho Rocha, Anne Carolinne Bezerra Perdigão, Nayara Santos de Oliveira, Fernanda Montenegro de Carvalho Araújo, Marcela Helena Gambim Fonseca, Giovanna Rolim Pinheiro Lima, Magda Moura de Almeida, Carlos Henrique Alencar, Wanderson Kleber de Oliveira, Luciano Pamplona de Góes Cavalcanti

**Affiliations:** 1 Universidade Federal do Ceará, Faculdade de Medicina, Programa de Pós-Graduação em Saúde Pública, Fortaleza, CE, Brasil.; 2 Centro Universitário Christus, Faculdade de Odontologia, Fortaleza, CE, Brasil.; 3 Centro Universitário Christus, Faculdade de Medicina, Fortaleza, CE, Brasil.; 4 Centro Universitário Christus, Faculdade de Biomedicina, Fortaleza, CE, Brasil.; 5 Fundação Oswaldo Cruz, Fortaleza, CE, Brasil.; 6 Secretaria de Saúde do Estado do Ceará, Fortaleza, CE, Brasil.; 7 Universidade Federal do Ceará, Faculdade de Medicina, Programa de Pós-Graduação em Patologia, Fortaleza, CE, Brasil.; 8 Supremo Tribunal Federal, Secretaria de Serviços Integrados de Saúde, Brasília, DF, Brasil.

**Keywords:** Covid-19, Survey, School, SARS-CoV-2

## Abstract

**Background:**

The school community was heavily impacted by the Covid-19 pandemic, especially with the long time of school closures. This study aimed to analyze the seroprevalence of SARS-CoV-2 antibodies and possible factors associated with seropositivity for COVID-19 in teachers and other school staff, and to estimate the fraction of asymptomatic individuals by sex and age group.

**Methods:**

We conducted a serological survey of SARS-CoV-2 infections. An analytical cross-sectional study was conducted in Fortaleza, Brazil. Teachers and other staff members from pre-schools to universities of higher education to were investigated.

**Results:**

A total of 1,901 professionals participated in the study, of which 1,021 were staff and 880 were teachers. The seroprevalence of SARS-CoV-2 was 8.0% (152/1901). In the seropositive group, 48.3% were asymptomatic. There was a predominance of women (68.4%); and, 47.1% of the participants were between 31 and 45 years old. There was an increase in prevalence with increasing age. An inverse relationship was found for education level: more professionals with less education tested positive for COVID-19. The presence of an infected person living in the same household was significantly associated with positive results for COVID-19 among the professionals.

**Conclusions:**

This is the first study to report the seroprevalence of IgG against SARS-CoV-2 in Brazilian educational staff after the first wave of the disease. In this study, the seroprevalence was much lower than that in the general population. During school reopening, a small fraction of school workers showed serologically detectable signs of SARS-CoV-2 exposure.

## INTRODUCTION

The detection and spread of an emerging respiratory disease is associated with a huge amount of uncertainty regarding its epidemiological and serological characteristics^1^. The novel coronavirus, SARS-CoV-2, emerged in Wuhan, China, in December 2019 and rapidly spread to other countries^2^ ^,^ ^3^. On March 11, 2020, the World Health Organization (WHO) declared the coronavirus disease (COVID-19) a pandemic^4^.

To reduce transmission speed, control measures were launched worldwide; thus, pubs, shopping malls, parks, and schools were closed to avoid social contact^5^ ^,^ ^6^.

Despite the reopening of several sectors of the economy, more than 100 countries did not schedule dates for the reopening of schools until May 2020^7^.

The state of Ceará in northeast Brazil was one of the first to confirm sustained SARS-CoV-2 transmission, and schools were closed on March 20, 2020. The state government of Ceará issued early guidance for safe operation through prevention, early detection, and control of COVID-19 in schools and other educational facilities^8^. Sectoral protocol N.18 mentions, among other requirements, that alcohol gel must be available in all rooms; a minimum of 1.5 meters of space should be kept between school desks; classes should be filled up to 35% capacity; and, students, teachers, and staff should mandatorily use masks. In addition, all teachers and staff were tested for COVID-19 by RT-PCR until one week before classroom return.

Available evidence suggests that children and adolescents may be less susceptible and present less severe disease than adults^9^. However, there are reports in the northeast of the systemic inflammatory syndrome in children during the COVID-19 pandemic^10^ ^,^ ^11^.

As SARS-CoV-2 is a new virus, its initial seroprevalence in the population is assumed to be negligible. Therefore, the surveillance of antibody seropositivity in specific populations can allow inferences regarding the extent of infection in this population and subsequent control measures. By the end of November 2020, the state of Ceará had reported 293.237 cases and 9.563 deaths^12^ ^,^ ^13^. 

The main objective of this study was to measure the seroprevalence of antibodies against anti-SARS-CoV-2 IgG antibodies, to identify factors associated with infection in teachers and other school staff to ascertain the cumulative population immunity, and to estimate the prevalence of asymptomatic infection by sex and age group after the first wave of the disease in Brazil. This is particularly important in the context of novel respiratory pathogens, such as SARS-CoV-2, and in the context of education.

## METHODS

We conducted a serological survey using chemiluminescence immunoassay for anti-SARS-CoV-2 immunoglobulin G (IgG) antibodies in 2,341 private school teachers and staff in Fortaleza, Brazil. In 2020, only private schools returned to classroom lessons five months after the first pandemic peak and two months after the schools reopened.

In this section, we describe the study location, sampling and recruitment approaches, specimen collection methods, antibody testing procedures, statistical methods, and ethical aspects.

### Design and study site

This was an analytical cross-sectional survey study with data collected between October and November 2020 in Fortaleza, Northeastern Brazil. 

### Study Participants and Sample Recruitment

We contacted schools through the union of private schools in Fortaleza (SINEPE-CE) to explain the research. The union, founded in 1943, represented the private schools in Fortaleza. The study was authorized, and the schools and their respective employees were invited to participate in the research. 

A Google Forms link was subsequently sent to all the schoolteachers, university professors, and staff. Individuals who clicked on the link were directed to a survey that provided information regarding the study. At the end of the questionnaire, blood sample collection was scheduled to be conducted at the school. Only individuals with participant IDs were allowed to enter the testing area. The education professionals who participated in the survey and provided blood samples received the results of their tests through a smartphone application.

Professionals from pre-schools to universities of higher education were invited.

### Laboratory diagnoses

The collected blood samples for serum separation were transported to a local laboratory where they were centrifuged at 2500 rpm for 10 min in an EVLAB apparatus (Macro EV model 04). Subsequently, they were frozen at -20 °C and transported to the Laboratory of Clinical Analysis of the Unichristus University Center (*Laboratório Escola de Análises Clínicas da Unichristus* (LEAC), in Portuguese) or to the laboratory of the *Fundação Oswaldo Cruz* (FIOCRUZ-CE) for testing. All samples were tested for IgG using the Abbott ARCHITECT SARS-CoV-2 test, a fully automated indirect immunoassay that detected antibodies directed to a recombinant SARS-CoV-2 nucleocapsid antigen. The assay showed a very high specificity (94.4%) and 100% sensitivity for samples collected after 14 days of symptom onset^14^ ^,^ ^15^. 

### Study variables and data analysis

The variables used in this study were demographic data: sex, age, educational level, family income, self-reported COVID-19 symptoms, and the presence of chronic diseases such as diabetes, hypertension, asthma, chronic kidney disease, and cancer. Other aspects were related to housing type (house or apartment), number of people living in the house, presence of basic sanitation, garbage collection, and water supply. Variables regarding working conditions included: work shift (morning, afternoon, or night), mode of transportation used, habits during the pandemic, and individual protection measures. We also investigated the class levels of the teachers, and, for the other employees, the sector in which they worked (secretary, administrative, canteen, concierge, and cleaning). In terms of ethnicity, we used a self-reported standard Brazilian skin color/ethnicity classification using five categories: white, black, brown, Asian, and indigenous. The “brown” or “*pardo*” category included individuals who self-reported having mixed ancestry.

### Statistical analysis

The data were exported to IBM SPSS Statistics for Windows version 20.0. The association of the frequency of serum conversion to COVID-19 with other variables was checked using Pearson chi-square test or Fisher exact test. Variables with p<0.200 were subjected to a multivariable logistic regression model. Adjusted odds ratios and 95% CIs were calculated to determine the factors independently associated with COVID-19-IgG seroconversion.

### Ethical aspects

The study was approved by CAAE 39691420.7.0000.5049.

## RESULTS

### Characteristics of the population

In total, 2,341 questionnaires were completed. After removing duplicates, 340 (14.5%) were excluded. After blood collection, another 100 (5%) were excluded due to insufficient material for testing. We then collected data from 1,901 professionals, including 1,021 employees and 880 teachers. Most of the teachers (34.2%) taught in elementary I; 24.0%, elementary II; 23.8%, higher education; and, 23.2%, preschools.

There was a predominance of women (68.4%), and 47.1% of the participants were between 31 and 45 years old (Table 1). Most participants lived in houses (63.1%) with up to three people (56.9%) (Table 1). Most of the interviewees reported the absence of persons diagnosed with COVID-19 at home (57.6%), and 68.4% reported knowing someone who had died from COVID-19. 

**TABLE 1:** Influence of socioeconomic factors on seroconversion in teachers and school employees during the first wave of the COVID-19 pandemic in the city of Fortaleza, Brazil**.**

Sociodemographic variables	Univariable Analysis				Multivariable logistic regression	
	Total	COVID-19 IgG		p-value	Adjusted OR (95%CI)	p-value
		Negative	Positive			
**Gender** ^¥^						
Female	1300 (68.4%)	1197 (68.5%)	103 (67.8%)	0.856	-	-
Male	600 (31.6%)	551 (31.5%)	49 (32.2%)		RC	-
**Age**						
Up to 30 years old	563 (29.6%)	522 (29.8%)	41 (27.0%)	0.002	RC	-
31 to 45 years old	896 (47.1%)	838 (47.9%)	58 (38.2%)		-	-
>45 years old	442 (23.3%)	389 (22.2%)	53 (34.9%)		2.39 (1.12-5.13)	0.025
**Race**						
White	634 (34.0%)	600 (35.0%)	34 (22.8%)	0.001	0.80 (0.44-1.46)	0.467
Brown	1024 (54.9%)	926 (54.0%)	98 (65.8%)		RC	
Black	145 (7.8%)	128 (7.5%)	17 (11.4%)		-	
Yellow	62 (3.3%)	62 (3.6%)	0 (0.0%)		-	
**Education level**						
Up to elementary school/High school	496 (26.1%)	445 (25.4%)	51 (33.6%)	0.078	RC	
University	753 (39.6%)	696 (39.8%)	57 (37.5%)		-	
Postgraduate	652 (34.3%)	608 (34.8%)	44 (28.9%)		-	
**Marital status**						
Married	887 (46.7%)	813 (46.5%)	74 (48.7%)	0.022	1.44 (0.73-2.84)	0.290
Single	751 (39.5%)	696 (39.8%)	55 (36.2%)		RC	
Divorced	124 (6.5%)	118 (6.7%)	6 (3.9%)		-	
Stable union	114 (6.0%)	103 (5.9%)	11 (7.2%)		-	
Widower	25 (1.3%)	19 (1.1%)	6 (3.9%)		-	
**Housing Type**						
House	1198 (63.1%)	1089 (62.3%)	109 (71.7%)	0.021	-	-
Apartment	702 (36.9%)	659 (37.7%)	43 (28.3%)		RC	-
**People living in the house**						
Up to 3	1060 (56.9%)	987 (57.6%)	73 (48.3%)	0.027	0.86 (0.47-1.57)	0.620
>3	804 (43.1%)	726 (42.4%)	78 (51.7%)		RC	
**Housing Conditions**						
It has basic sanitation	1700 (89.5%)	1566 (89.6%)	134 (88.2%)	0.582	RC	
It has garbage collection	1890 (99.5%)	1740 (99.5%)	150 (98.7%)	0.161	0.98 (0.50-10.51)	1.000
It has piped water	1895 (99.7%)	1745 (99.8%)	150 (98.7%)	0.008	3.00 (0.30-13.13)	1.000

Subtitle: *p<0.05, Fisher exact test or Pearson chi-square test; ^¥^Not everyone filled in this information; **OR:** odds ratio; **95% CI:** 95% confidence interval of adjusted OR; **RC:** reference category in the multivariate analysis.

The most used mode of transportation to work was personal cars (45.9%), followed by public transportation (31.9%). The majority (74.1%) reported to have avoided face-to-face activities during the pandemic. They stayed at their home offices, and 60.0% of them reported to leave home only to go to the marketplace and to the pharmacy (40.0%) (Tables 2 and 3).

**TABLE 2:** Influence of professional profile on seroconversion in school teachers and employees during the first wave of the COVID-19 pandemic in the city of Fortaleza, Brazil.

Professional profile	Univariable Analysis				Multivariable logistic regression	
	Total	COVID-19 IgG		p-value	Adjusted OR (95%CI)	p-value
		Negative	Positive			
**Function**						
Teacher	880 (46.3%)	815 (46.6%)	65 (42.8%)	0.363	-	-
Collaborator	1021 (53.7%)	934 (53.4%)	87 (57.2%)		RC	-
**Shift work**						-
Morning	1615 (85.0%)	1494 (85.4%)	121 (79.6%)	0.054	0.66 (0.31-1.38)	0.268
Afternoon	1358 (71.4%)	1252 (71.6%)	106 (69.7%)	0.629	-	-
Night	351 (18.5%)	323 (18.5%)	28 (18.4%)	0.989	RC	-
**Works quantity shifts**					-	-
<1	2 (0.1%)	2 (0.1%)	0 (0.0%)	0.311	-	-
1	610 (32.1%)	551 (31.5%)	59 (38.8%)		-	-
2	1153 (60.7%)	1070 (61.2%)	83 (54.6%)		-	-
3	136 (7.2%)	126 (7.2%)	10 (6.6%)		RC	-
**Time spent teaching in the classroom** (in years)^€^					-	-
< 6	199 (23.0%)	186 (23.2%)	13 (20.3%)	0.092	RC	-
6-10	238 (27.5%)	221 (27.6%)	17 (26.6%)		-	-
11-20	247 (28.5%)	234 (29.2%)	13 (20.3%)		-	-
>20	182 (21.0%)	161 (20.1%)	21 (32.8%)		0.99 (0.44-2.22)	0.974
**Transportation used for work**					-	-
Bicycle	52 (2.7%)	46 (2.6%)	6 (3.9%)	0.235	-	-
Hitchhiking with co-workers	87 (4.6%)	81 (4.6%)	6 (3.9%)		-	-
Car alone	873 (45.9%)	814 (46.6%)	59 (38.8%)		RC	-
Motorcycle with co-worker	26 (1.4%)	24 (1.4%)	2 (1.3%)		-	-
Motorcycle alone	101 (5.3%)	87 (5.0%)	14 (9.2%)		-	-
Collective Transportation	606 (31.9%)	553 (31.6%)	53 (34.9%)		-	-
Other	155 (8.2%)	143 (8.2%)	12 (7.9%)		-	-
**Professional performance**					-	-
Preschool	204 (23.2%)	186 (22.8%)	18 (27.7%)	0.371	RC	-
Elementary I	301 (34.2%)	274 (33.6%)	27 (41.5%)	0.195	1.23 (0.65-2.30)	0.523
Fundamental II	211 (24.0%)	200 (24.5%)	11 (16.9%)	0.166	0.89 (0.40-1.98)	0.767
High School	188 (21.4%)	176 (21.6%)	12 (18.5%)	0.553	-	-
Higher Education	209 (23.8%)	197 (24.2%)	12 (18.5%)	0.296	-	-
Post-graduation	72 (8.2%)	67 (8.2%)	5 (7.7%)	0.881	-	-
Technical courses	19 (2.2%)	18 (2.2%)	1 (1.5%)	0.721	-	-
**Was excluded from face-to-face activities in the pandemic**					-	-
No	493 (25.9%)	460 (26.3%)	33 (21.7%)	0.214	RC	-
Yes	1407 (74.1%)	1288 (73.7%)	119 (78.3%)		-	-
Has health insurance	1462 (76.9%)	1356 (77.6%)	106 (69.7%)	0.028	0.45 (0.18-1.17)	0.102
**Practices physical activity**						
No	930 (49.0%)	843 (48.3%)	87 (57.2%)	0.034	0.50 (0.28-0.92)	0.025
Yes	969 (51.0%)	904 (51.7%)	65 (42.8%)		RC	

Subtitle: *p<0.05, Fisher exact test or Pearson chi-square test; ^¥^ Not everyone provided this information; **OR:** odds ratio; **95% CI:** 95% confidence interval of adjusted OR; **RC:** reference category of multivariable analysis.

**TABLE 3:** Influence of daily routine on seroconversion in school teachers and employees during the first wave of the COVID-19 pandemic in the city of Fortaleza, Brazil.

Daily Routine	Univariable Analysis				Multivariable logistic regression		
	Total	COVID-19 IgG		p-value	Adjusted OR (95%CI)	p-value
		Negative	Positive			
**In your home, someone had COVID19**						
No	1095 (57.6%)	1044 (59.7%)	51 (33.6%)	<0.001	5.58 (3.02-10.30)	<0.001^¥^
I don't know	458 (24.1%)	418 (23.9%)	40 (26.3%)		-	
Yes	348 (18.3%)	287 (16.4%)	61 (40.1%)		RC	
**Do you know someone who had COVID-19?**						
No	194 (10.2%)	176 (10.1%)	18 (11.8%)	0.487	-	-
Yes	1707 (89.8%)	1573 (89.9%)	134 (88.2%)		RC	
**Do you know anyone who died from COVID-19?**						
No	600 (31.6%)	556 (31.8%)	44 (28.9%)	0.470	-	-
Yes	1301 (68.4%)	1193 (68.2%)	108 (71.1%)		RC	
**Did you succeed in social isolation?**						
Practically isolated from the world	312 (16.5%)	293 (16.8%)	19 (12.5%)	0.266	-	-
Very little	67 (3.5%)	61 (3.5%)	6 (3.9%)		-	
Not much	35 (1.8%)	30 (1.7%)	5 (3.3%)		-	
More or less	345 (18.2%)	311 (17.8%)	34 (22.4%)		-	
Quite	1137 (60.0%)	1049 (60.1%)	88 (57.9%)		RC	
**How was your routine during social isolation?**						
Staying at home all the time	295 (15.6%)	279 (16.1%)	16 (10.6%)	0.461	-	-
Going out only for essential things, like buying food or a pharmacy	1265 (67.1%)	1160 (66.9%)	105 (69.5%)		-	
Going out once in a while to buy food and stretching legs	114 (6.0%)	103 (5.9%)	11 (7.3%)		-	
Going out every day for some activity	39 (2.1%)	35 (2.0%)	4 (2.6%)		-	
Going out every day, all day, to work or for other regular activities	173 (9.2%)	158 (9.1%)	15 (9.9%)		RC	
**Visiting routine in your home**						
Only who lives in the house and nobody else	911 (49.2%)	845 (49.7%)	66 (43.7%)	0.433	-	-
Some close relatives visit once or twice a week	757 (40.9%)	689 (40.6%)	68 (45.0%)		-	
Friends, distant relatives, or others who visit once or twice a week	89 (4.8%)	80 (4.7%)	9 (6.0%)		-	
Some close relatives visit almost every day	79 (4.3%)	71 (4.2%)	8 (5.3%)		-	
Friends, distant relatives, or others who visit every day	14 (0.8%)	14 (0.8%)	0 (0.0%)		RC	
**Do you consider that the following protect you against COVID-19**						
Wearing a mask every time you leave the house	1796 (94.5%)	1651 (94.4%)	145 (95.4%)	0.605	RC**	-
Staying at home and avoiding contact with other people	1207 (63.5%)	1110 (63.5%)	97 (63.8%)	0.931	RC**	-
Cleaning your hands with alcohol gel	1557 (81.9%)	1427 (81.6%)	130 (85.5%)	0.227	RC**	-
Avoiding people while outside the home	1335 (70.2%)	1234 (70.6%)	101 (66.4%)	0.288	RC**	-
Washing your hands frequently	1818 (95.6%)	1672 (95.6%)	146 (96.1%)	0.792	RC**	-
Not putting your hands in your mouth, nose, or eyes	1604 (84.4%)	1475 (84.3%)	129 (84.9%)	0.862	RC**	
Taking chloroquine	45 (2.4%)	42 (2.4%)	3 (2.0%)	0.739	RC**	
Being young	30 (1.6%)	26 (1.5%)	4 (2.6%)	0.277	RC**	

Subtitle: *p<0.05, Fisher exact test or Pearson chi-square test; ^¥^Considering only those who declared yes or no; **RC:** reference category of multivariable analysis; **RC: reference category of multivariable analysis is professionals who do not respond to this item.

### Characteristics of positive cases of COVID-19

IgG antibodies were detected in 152 of the 1,901 samples, with a positive seroprevalence of 8.0% (95% CI: 6.8-9.3). Among the seropositive participants, 48.3% did not report prior COVID-19-like illnesses. 

An increase in the seroprevalence was observed with increasing age. Individuals older than 45 years showed 12.0% positivity (PR=1.63; 95%CI: 1.11-2.41). The seroprevalence in the brown and black races was 9.6% (PR=1.97; 95%CI:1.35-2.88) and 11.7% (PR=2.18; 95%CI: 1.25-3.80), respectively, and it was significantly higher in them than those who declared themselves white (5.4%). 

The seropositivity among the staff was slightly higher (8.5%; 95%CI: 7.0-10.2) as compared to that in teachers (7.4%; 95%CI: 5.9-9.4), but without a significant difference (p=0.363). 

Contrastingly, for teachers, positivity for COVID-19 IgG antibodies was higher among those with more than 20 years of classroom teaching experience (11.5%; 95%CI: 7.6-17.6). Teachers working in preschool classes and elementary I showed 8.8% (95%CI: 5.6-13.6) and 9.0% (95%CI: 6.2-12.8) positivity, respectively, which was higher than the average positivity of the other classes (5.9%; 95%CI: 4.3-7.9). 

It was also observed that teachers excluded from the present classroom activities showed no significant difference in positivity for COVID-19 (p=0.214) (Table 2). The presence of infected persons at home was significantly associated with positivity for COVID-19 among professionals (PR=3.76; 95%CI: 2.65-5.35). 

Social isolation was declared as intense by 312 professionals, and this group had a mean prevalence of positivity of 6.1% (95%CI: 3.9-9.4), lower than that in professionals who did not maintain isolation at the same intensity (8.4%; 95%CI: 7.1-9.9). This was also observed in the social isolation routine, in which those who went out (8.5%; 95%CI: 7.2-9.9) and those who received more visitors (9.0%) (95%CI: 7.4-11.7) had higher positivity rates than those who stayed at home all the time (5.4%; 95%CI: 3.3-8.7).

The most prevalent symptoms among participants with positive tests who reported prior COVID-19-like illnesses were loss of smell, loss of taste, fever, body pain, and cough, with percentages higher than 25%, with a statistically significant difference from those professionals with the same symptoms but showing negative tests. The only symptom that was not statistically significant was headache, with only seven reports (p=0.078). Among the symptomatic patients, six different groups of medications were prescribed, and azithromycin (40.3%), dipyrone (35.1%), ivermectin (27.7%), and paracetamol (25.5%) were notable. However, none of the medications used was associated with COVID-19 symptoms (Table 4). 

**TABLE 4:** Perceptions of people who had symptoms regarding seroconversion in teachers and school employees during the first wave of the COVID-19 pandemic in the city of Fortaleza, Brazil.

Perception of the symptomatic	Total	IgG COVID-19		p-Value
		Negative	Positive	
**Symptoms**				
Chills	115 (6.1%)	90 (5.1%)	25 (16.4%)	<0.001
Diarrhea	79 (4.2%)	65 (3.7%)	14 (9.2%)	0.001
Difficulty breathing	104 (5.5%)	77 (4.4%)	27 (17.8%)	<0.001
Headache	47 (2.5%)	40 (2.3%)	7 (4.6%)	0.078
Sore throat	62 (4.0%)	56 (3.9%)	6 (4.9%)	<0.001
Pain in the body	189 (9.9%)	145 (8.3%)	44 (28.9%)	<0.001
Fever	209 (11.0%)	159 (9.1%)	50 (32.9%)	<0.001
Loss of sense of smell	223 (11.7%)	164 (9.4%)	59 (38.8%)	<0.001
Loss of taste	233 (12.3%)	176 (10.1%)	57 (37.5%)	<0.001
Cough	173 (9.1%)	134 (7.7%)	39 (25.7%)	<0.001
**If you have had symptoms. have taken any medication**				
No	73 (17.4%)	63 (18.6%)	10 (12.3%)	0.180
Paracetamol	107 (25.5%)	83 (24.6%)	24 (29.6%)	0.347
Azithromycin	169 (40.3%)	129 (38.2%)	40 (49.4%)	0.065
Hydroxychloroquine/chloroquine	26 (6.2%)	18 (5.3%)	8 (9.9%)	0.127
Dipyrone	147 (35.1%)	116 (34.3%)	31 (38.3%)	0.503
Ivermectin	116 (27.7%)	89 (26.3%)	27 (33.3%)	0.206
Zinc	50 (12.1%)	37 (11.1%)	13 (16.3%)	0.202
**It is possible to avoid the disease**				
I don't know	182 (9.6%)	166 (9.5%)	16 (10.5%)	0.893
No	104 (5.5%)	94 (5.4%)	10 (6.6%)	
Maybe	597 (31.4%)	550 (31.4%)	47 (30.9%)	
Yes	1018 (53.6%)	939 (53.7%)	79 (52.0%)	
**Looked for a doctor**	156 (70.0%)	115 (67.3%)	41 (78.8%)	0.110
**Had COVID-19**	409 (21.5%)	328 (18.8%)	81 (53.3%)	<0.001
**Laboratory confirmation COVID-19**	76 (34.9%)	56 (33.7%)	20 (38.5%)	0.533
**You think you got COVID-19 from someone you know**	119 (53.4%)	86 (50.3%)	33 (63.5%)	0.096

Subtitle: *p<0.05, Fisher's exact test or Pearson's chi-square test.

In an adjusted analysis, the chance of positivity among those aged >45 years was 2.39 times higher (95%CI: 1.12-5.13; p=0.025), and seroconversion was 2.00 times lower in those who did not perform physical activity (95%CI: 1.09-3.57; p=0.025). For those who had patients with COVID-19 at home, the chance of positivity was 5.58 times higher (95%CI: 3.03-10.3; p<0.001). Regarding symptoms, difficulty in breathing and loss of smell were notable, which were 4.04 and 4.12 times higher among those who showed positive results (p<0.001), respectively. 

Employees who used public transportation to attend school showed higher positivity rates. On the other hand, lower positivity rates were observed in teachers who worked only one shift, commuted alone in their cars, and had health insurance (Table 5).

**TABLE 5:** Influence of professional profile on seroconversion in school teachers and employees during the first wave of the COVID-19 pandemic in the city of Fortaleza, Brazil**.**

Professional Profile	Total	Function		p-Value
		Teacher	Collaborator	
**Shift work**				
Morning	1615 (85.0%)	738 (83.9%)	877 (85.9%)	0.216
Afternoon	1358 (71.4%)	541 (61.5%)	817 (80.0%)	<0.001
Night	351 (18.5%)	185 (21.0%)	166 (16.3%)	0.008
**Number of shifts you work**				
<1	2 (0.1%)	1 (0.1%)	1 (0.1%)	<0.001
1	610 (32.1%)	359 (40.8%)	251 (24.6%)	
2	1153 (60.7%)	455 (51.7%)	698 (68.4%)	
3	136 (7.2%)	65 (7.4%)	71 (7.0%)	
**Time spent teaching in the classroom£**				
Up to 5 years	199 (23.0%)	199 (23.0%)	0 (0.0%)	0.450
6-10 years old	238 (27.5%)	237 (27.4%)	1 (100.0%)	
11-20 years old	247 (28.5%)	247 (28.6%)	0 (0.0%)	
>20 years	182 (21.0%)	182 (21.0%)	0 (0.0%)	
**Transportation used to go to work**				
Bicycle	52 (2.7%)	6 (0.7%)	46 (4.5%)	<0.001
Hitchhiking with co-workers	87 (4.6%)	47 (5.3%)	40 (3.9%)	
Car alone	873 (45.9%)	601 (68.3%)	272 (26.7%)	
Motorcycle with co-worker	26 (1.4%)	3 (0.3%)	23 (2.3%)	
Motorcycle alone	101 (5.3%)	27 (3.1%)	74 (7.3%)	
Collective Transportation	606 (31.9%)	123 (14.0%)	483 (47.4%)	
Other	155 (8.2%)	73 (8.3%)	82 (8.0%)	
**Was removed from classroom activities during quarantine**				
No	493 (25.9%)	232 (26.4%)	261 (25.6%)	0.701
Yes	1407 (74.1%)	648 (73.6%)	759 (74.4%)	
**Has health insurance**	1462 (76.9%)	789 (89.8%)	673 (65.9%)	<0.001
**Works in more than one institution**	245 (12.9%)	213 (24.2%)	32 (3.1%)	<0.001

Subtitle: *p<0.05, Fisher's exact test or Pearson's chi-square test; ^£^For faculty only.

## DISCUSSION

This study was the first large-scale prevalence study conducted among educational workers in Brazil immediately after the first wave of the disease. This study was the first to measure the IgG antibody response to SARS-CoV-2 in a school community exposed to the virus. In our study, the immune response related to previous SARS-CoV-2 infections was < 10.0%. The seropositivity was lower than that of the general population (15.53%) when tested during the same period in the city of Fortaleza^13^. 

Seroprevalence studies help understand the likelihood of asymptomatic infections. Among our participants who tested seropositive, 48.3% reported no prior COVID-19-like illnesses. This finding suggests that a significant proportion of patients with COVID-19 were asymptomatic. It is likely that these individuals did not self-isolate when infected, and they continued to spread the disease to other people. 

A population-based survey conducted in another state in the northeast region of Brazil showed that the seroprevalence of total antibodies against SARS-CoV-2 was 40.4%^16^, much higher than that found in this study. A possible explanation for this low prevalence is that some teachers were working remotely at the time of the research. Another explanation for this difference is that we did not perform IgM antibody detection. 

Prevalence studies conducted during the first wave of the disease reported varying results owing to the population studied, sampling, and type of laboratory test used^17^ ^,^ ^18^. In this study, most factors associated with SARS-CoV-2 infection were identified outside the workplace, suggesting that current infection prevention strategies within schools can be effective in preventing transmission in the workplace.

These assays detected the presence of antibodies, but neutralization assays would be fundamental and complementary in determining the functional role of antibodies in immune protection^18^. 

This study showed that almost half of the IgG-positive cases were asymptomatic. Anosmia and ageusia predominated among the symptomatic cases. A study conducted by a private laboratory in the city of Fortaleza showed that 18.8% of the reported cases were asymptomatic^19^. Among those who reported symptoms, the most frequently reported symptoms were headache (36.40%), cough (29.62%), weakness (29.68%), and fever (27.42%). 

The percentage of asymptomatic patients was consistent with that reported in the literature^20^. Therefore, it is important to continue to reinforce the need for the correct use of proper face masks by professionals^21^, as asymptomatic cases may suggest a lower antibody response and titers decrease more quickly^22^. However, due to the large number of asymptomatic cases or mild infections and the difficulty of access to laboratory diagnosis in developing countries such as Brazil, the available data of laboratory-confirmed cases do not capture the true extent of virus spread. Therefore, the serological detection of specific antibodies against SARS-CoV-2 can be used to better estimate the true number of infections. 

The current evidence shows that schools have not evolved into silent *hotspots* of SARS-CoV-2 transmission. This is especially important as there are severe adverse effects of prolonged school closure, especially on populations that are more socially vulnerable^23^ ^,^ ^24^. Furthermore, we cannot fail to mention that in socially disadvantaged contexts, even with school closure, social contacts and non-school encounters continue^25^, thus reducing the potential benefit of school closure.

Our findings showed that those with higher education had a lower chance of a previous infection. This probably occurred because those with higher education had more access to information and, consequently, to disease prevention measures, such as social distancing, use of masks and chamging them within the period established by the competent bodies, use of *face shields* as physical barriers, and respiratory etiquette, among others recommended by the Brazilian Ministry of Health^12^. Furthermore, the greater adherence and compliance to safety rules by education professionals is notable.

Even in the scenario of high SARS-CoV-2 transmission, the spread within schools was very low. Modelling studies on the effect of school closure often rely on strong theoretical assumptions that do not easily adequately control for important confounders because of their ecological nature and, despite being interesting from a scientific point of view, they should not replace studies based on prospectively collected data. Schools should not be closed for a prolonged period, as they lead to overall harmful consequences on health, society, and the economy, in addition to increasing the existing inequalities between public and private education networks^26^ ^-^ ^28^. As evidence for COVID-19 evolves, there is heightened awareness of the disproportionate impact on the school community resulting from the closure of schools and an intensified call to reopen schools safely^29^.

The need to respond to the pandemic has led to the closure of school buildings across the country, with little time to ensure continuity of instruction or to create a framework for deciding when and how to reopen schools^30^. This was the first time in our country that all schools were closed for so long, which provided a unique opportunity to assess the influence of school closure not only on schoolchildren but also on the economy. A recent study conducted in the Gaza Strip highlighted the profound economic and social consequences^31^. As a result, parents, schools, and social organizations need to pay more attention to the psychological state of students, especially those in elementary school who have remained out of school for long^32^ ^,^ ^33^. Furthermore, our study involved professionals from private schools. Even though most of them, especially teachers, were public school teachers, they may present a different socioeconomic context than those who worked exclusively in public schools.

The presence of a person with a confirmed diagnosis of COVID-19 in the home increased the chance of testing positive by more than fivefold (5.58). The attack rates among family members were higher, and this finding reinforces previous literature that indicated the importance of isolation of close contacts and the need for mask use and intradomiciliary care^34^. 

Our study showed that the population of teachers and employees of private institutions aged over 45 years showed more than twice as high positivity (2.39) compared to the younger population. These findings corroborate the literature regarding age, indicating a higher positivity among older people^35^. 

No significant sex-related difference was observed, although women represented 68% of the sample. Moreover, there were no differences in housing conditions, job function, shift, time or duration of teaching, type of transportation, area of activity in the school, and whether they were away from classroom activities. There was a higher incidence of positive COVID-19 cases in the brown and black populations. The reality of socioeconomic vulnerability is also associated with a housing issue, wherein households limited to a smaller geographic space having a high number of household contacts (above three people) worsen the spread of SARS-COV-2^35^ ^,^ ^37^. The pandemic presented deep racial and social disparities, with more severe consequences in brown and black people^29^ ^,^ ^38^. 

In the face of the unprecedented global health crisis in recent decades, public health authorities need seroprevalence data to estimate the exposure of the most vulnerable groups, especially in developing countries where access to molecular diagnostics is limited. These prevalence estimates should be used to calibrate the projections of the epidemic and its actual mortality rate. Several lessons have been learned over these months, and we hope that educators and decision-makers will be better prepared to act promptly in future education crises involving interrupted classroom instruction. Currently, there is broad agreement that school closures involve heavy burdens on students, parents, and the economy with profound equity implications^39^.

Future studies should investigate the structural conditions of the school, such as the size of the physical space of the institution and the capacity of the sanitary facilities, which may favor or prevent the spread of the virus. Governments should reinforce, as soon as possible, policies that decrease transmission in the community and implement control measures within schools so that they can simultaneously address both the health crisis represented by COVID-19 and the adverse consequences of prolonged school closures^36^ ^,^ ^40^. Variation across schools in this condition is an additional complication in ensuring the health of students and staff at schools. To reopen safely, schools are encouraged to ensure ventilation and air filtration, clean surfaces frequently, provide facilities for regular handwashing, and provide space for physical distancing. 

During the first epidemic wave, many countries included school closures among the measures implemented to limit viral transmission^41^. Part of this decision was based on the experience of influenza transmission in schoolchildren^42^ ^,^ ^43^. With the circulation of new variants, it is critical to assess the risk of viral circulation among students and their teachers in schools^33^ because, to the best of our knowledge, secondary transmission of SARS-CoV-2 in school settings has been limited, as reported in Australia, Ireland, and France^44^ ^-^ ^46^. It is also important to better understand the extent of infection among teachers and its role in transmission within the school, given the likely negative effects of school closures on educational performance and economic outcomes^26^ ^,^ ^33^. Future decisions regarding school closures during the pandemic should give greater weight to the potential effects of school closure on children’s health^26^.

A limitation that must be reported is that the tests may result in false negatives for very recent infections, especially in the first two weeks after infection; therefore, this prevalence would reflect the infection levels one or two weeks prior to the date of the survey. In addition, it is important to note that, at the time of the study, the expression of the new strains was quite limited, and we had not yet isolated P.1., which became predominant in February 2021^47^. The use of online questionnaires and the convenience of sample size may have led to a bias in the results due to the interest of the persons participating in the study; however, we believe that given the high interest in the interviewees in taking the test for COVID-19, and given that the test was necessary for the return to work by the government of Ceará, this bias was minimized. In addition, the data collection of this study was carried out in a short period of time due to the need to obtain results that would enable an assessment of the association of the health situation of the employees with COVID-19. Furthermore, this study was conducted with professionals from private schools, and that its results must be extrapolated with restrictions to professionals from public schools, given the different working conditions of these professionals.

Therefore, in this new scenario, and considering the positive results of the measles vaccination^48^ ^,^ ^49^, it is essential to encourage vaccination not only for teachers and adolescents but also for the pediatric population. Without a doubt, schools need to reopen safely so that they can better serve students, families, and communities that depend on them.
